# Ten-Year Durability of Hypothalamic Deep Brain Stimulation in Treatment of Chronic Cluster Headaches: A Case Report and Literature Review

**DOI:** 10.7759/cureus.47338

**Published:** 2023-10-19

**Authors:** Aaradhya Pant, Farrokh Farrokhi, Katie Krause, Maria Marsans, John Roberts

**Affiliations:** 1 Neurosurgery, Virginia Mason Medical Center, Seattle, USA; 2 Neurological Surgery, Virginia Mason Medical Center, Seattle, USA; 3 Neurology, Virginia Mason Medical Center, Seattle, USA

**Keywords:** long-term outcome, chronic cluster headache, hypothalamus, deep brain stimulation, case report

## Abstract

Chronic cluster headache (CCH) is a debilitating primary headache that causes excruciating pain without remission. Various medical and surgical treatments have been implemented over the years, yet many provide only short-term relief. Deep brain stimulation (DBS) is an emerging treatment alternative that has been shown to dramatically reduce the intensity and frequency of headache attacks. However, reports of greater than 10-year outcomes after DBS for CCH are scant. Here, we report the durability of DBS in the posterior inferior hypothalamus after 10 years on a patient with CCH. Our patient experienced an 82% decrease in the frequency of headaches after DBS, which was maintained for over 10 years. The side effects observed included depression, irritability, anxiety, and dizziness, which were alleviated by changing programming settings. In the context of current literature, DBS shows promise for long-term relief of cluster headaches when other treatments fail.

## Introduction

Cluster headache is a debilitating primary headache belonging to the group of trigeminal autonomic cephalalgias [1]. Its prevalence is less than 1% and affects more men than women [2]. In episodic cluster headaches, headache attacks occur for weeks or months, followed by periods of remission lasting more than three months. In the chronic form, daily headache attacks occur without remission [2].

To date, numerous preventative measures to treat chronic cluster headaches (CCH) have been implemented. These include treatments such as suboccipital steroid injections, lithium, verapamil, warfarin, and melatonin [3-7]. Preventative surgical treatments include sphenopalatine ganglion lesioning, occipital nerve stimulation, and deep brain stimulation (DBS) [6,7].

DBS was first reported as a treatment for chronic cluster headaches after May et al. found activation and structural abnormality of the ipsilateral inferior hypothalamus using positron emission tomography and voxel-based morphometry [8-9]. Subsequently, Leone et al. led the first attempt to treat CCH with DBS in 2001 [10]. Their seminal discoveries have led to numerous reports on the clinical approach and efficacy of DBS of the tegmentum [11-13] and the hypothalamus [14-39] on treating chronic cluster headaches.

While DBS has been shown to reduce headache attack frequency in the short term, only one report has followed patients for more than 10 years [40]. Here, we add to the extant literature and report the history of a patient with posterior inferior hypothalamic stimulation for CCH after 11 years of follow-up.

## Case presentation

Our patient was a 40-year-old male at the time of presentation who had suffered from medically refractory right-sided cluster headaches since the age of 12, without remission. The headache attacks occurred 2-3 times per day, lasting between 15 minutes to 2 to 4 hours, with a pain rating of 10 out of 10. They were associated with nasal congestion, epiphora and ptosis, and increased with activity and stress. He described his headache quality as throbbing, aching, and stabbing with pressure and a dull sense, primarily in the orbital and temporal region. Functionally, these headaches prevented reliable participation in his professional and social activities. His MRI showed no intracranial pathology. These findings are consistent with the ICHD-3 classification of chronic cluster headache [1].

Several medications were trialed including acetaminophen, acetylsalicylic acid, naproxen, ibuprofen, indomethacin, dihydroergotamine, naratriptan, frovatriptan, zolmitriptan, eletriptan, almotriptan, propranolol, nadolol, nortriptyline, divalproex sodium, topiramate, levetiracetam, pregabalin, oxcarbazepine, multiple combination caffeine-based medications, as well as lidocaine nasal spray. Mild improvement in pain severity was noted with sumatriptan, rizatriptan, verapamil, oxygen, methadone, and morphine. The patient also underwent surgical treatments including sinus surgery ipsilateral to CH and sphenopalatine ganglion lesioning. The sphenopalatine ganglion lesioning resulted in transient reduction of headaches over three years, but repeat attempts did not provide sustained clinical improvement. For pain management, the patient has been taking methadone and oxycodone.

Due to the debilitating nature of his condition and exhaustion of all available medical and surgical treatments, DBS therapy was considered. The patient subsequently underwent DBS surgery of the posterior inferior hypothalamus.

Procedure details

**Figure 1:**
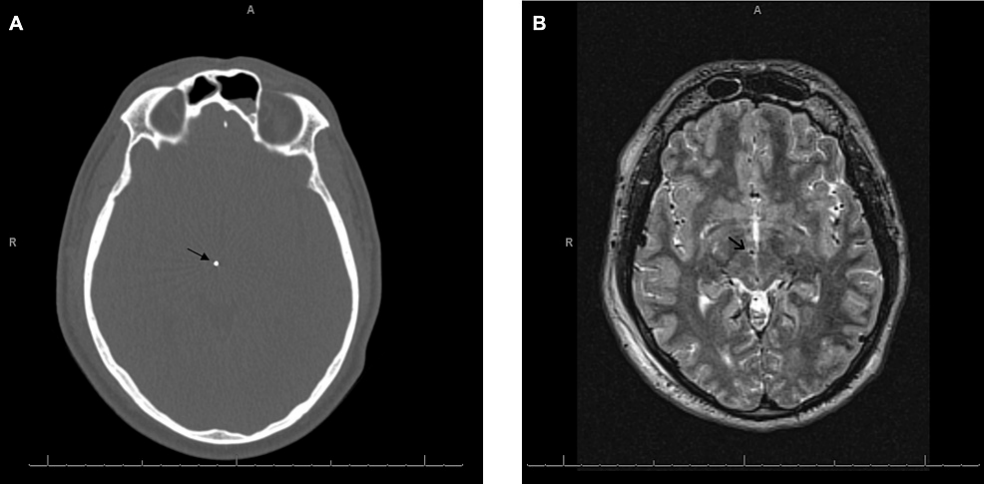
(A) CT and (B) MRI scans post-DBS surgery. The location of electrodes in the posterior inferior hypothalamus is shown with a black arrow. DBS: Deep brain stimulation

The stereotactic coordinates were determined by fusing a localized CT scan with the pre-operative targeting MRI. The operation was performed using a Cosman-Roberts-Wells (CRW) frame. Stimulation was performed using the Medtronic Activa SC pulse generator. The recording microelectrode was placed with its tip 10 mm above the right posterior inferior hypothalamic target, which was set at 2 mm lateral, 3 mm posterior, and 5 mm inferior to the middle commissural point. The patient had no adverse effects and could tolerate amplitudes as high as 5 volts in this location. CT (Figure 1A) and MRI (Figure 1B) post electrode placement are shown.

Long-term results

**Figure 2:**
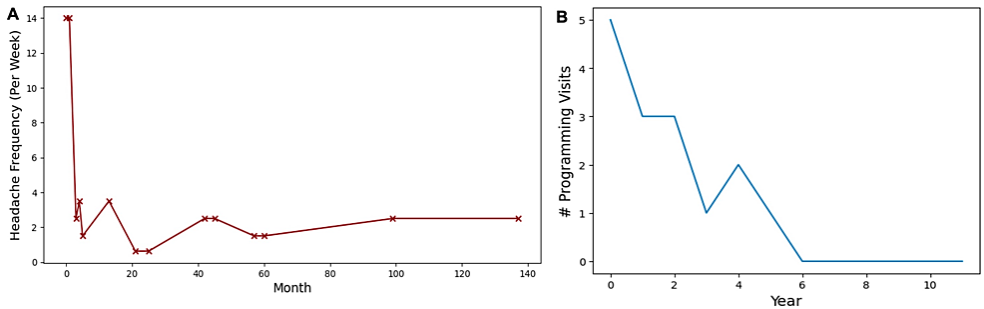
Change in headache frequency (A) and number of programming visits per year (B). (A) Change in headache frequency over time. Month 0 corresponds to headache frequency prior to deep brain stimulation (DBS) implant. (B) Number of programming visits per year. Year 0 corresponds to the year of DBS implant.

Over the 11 years following DBS implantation, the patient’s headache frequency decreased by 82% from 14 per week to 2.5 per week (Figure 2A). No improvement was noticed in the month after surgery but reduced dramatically to 2.5 per week after three months. However, over time the patient experienced side effects such as depression, irritability, anxiety, and dizziness with certain programming settings. Headache frequency reached its minimum (1.5 per week) around 20 months after DBS implantation, but gradually returned to 2.5 per week. Changes in the duration or severity of headache attacks for this patient were not recorded in clinical notes.

The patient had a pre-existing anxiety disorder, and he continued to experience anxiety during high-amplitude stimulation. After 2.5 years, the patient was prescribed sertraline to manage his anxiety. Furthermore, to avoid anxiety, the patient would leave DBS to below 1V or turn it off entirely, then increase the amplitude to 4V to abort an oncoming headache.

The patient visited the clinic 15 times over a five-year period to fine-tune a deep brain stimulation (DBS) program that effectively reduced their pain while minimizing side effects. The final optimized program used a method called "interleaving." In this setup, the implanted pulse generator (the "case") served as the positive electrical pole. The system alternated the negative electrical pole between two specific points in the brain, referred to as electrodes 1 and 2. By switching back and forth between these two setups, the program was able to better manage the patient's symptoms. The electrical settings for this optimized program were a pulse width of 60 milliseconds, a frequency of 80Hz, and an amplitude range between 0 and 3 volts.

The patient continued his use of methadone and oxycodone, which had been highly variable in the years prior to DBS placement with no sustained effect. Methadone was increased from 45 mg to 90 mg in morphine equivalent dose (MED), while oxycodone was maintained at 60 mg MED. This daily dosing regimen remained stable after DBS with sustained effect. The patient also used eletriptan or a sumatriptan injector, but not both, to abort headaches. He reports a reduction of usage of abortive dosing from 2-3 events per week pre-DBS to 1-2 events per month after DBS.

Patient perspective

The patient confirmed that prior to DBS surgery, he had no relief from headaches since the age of twelve. He mentioned that he is no longer on disability after 15 years, has returned to work, and that he has “got [his] life back”. The patient also reported some adverse effects such as irritability and anxiety, which he avoids by occasionally turning the device off. The patient encourages others with his condition to learn more about DBS.

## Discussion

The American Headache Society (AHS) recommends the following preventative treatments for reducing the frequency of chronic cluster headache attacks: suboccipital steroid injection, lithium, verapamil, warfarin, and melatonin [7]. However, DBS is currently listed as being probably ineffective in providing relief for chronic cluster headache.

There is increasing evidence for DBS in treating chronic cluster headaches. A randomized placebo-controlled double-blind trial by Fontaine et al. in 2009 showed no significant improvement compared to sham stimulation in the randomized phase of the study [41]. However, in the 10-month open phase, the mean weekly attacks decreased by 48.4%, with more than 50% of patients showing greater than 50% improvement. The small sample size of 12 patients, the short randomization phase, and the lack of parameter optimization during the randomized phase are important limitations to consider before determining the efficacy, or lack thereof, of DBS.

Pooled analyses of DBS in treating CCH reveal positive outcomes. A meta-analysis of 40 patients revealed a 75% response rate, defined as the percentage of patients having greater than 50% reduction in headache frequency. These patients had a mean reduction of 77% in headache frequency after DBS over an average follow-up of 44 months [38]. Another recent meta-analysis reported 108 cases with follow-up periods ranging from 1 to 144 months [42]. They report that 70% of patients achieved improvement in headache attack frequency and intensity after DBS implantation. Furthermore, Membrilla et al. report a pooled response rate of 77.0% with DBS for CCH [6].

Two studies have followed cluster headache patients after DBS for longer than five years. Piacentino et al. followed four patients five years after DBS [35]. They report a substantial decrease in the frequency of attacks and pain intensity and an increase in perceived quality of life and health status. Leone et al. report greater than 10-year outcomes of DBS for six patients, with ages ranging from 30 to 63 years [40]. Five of the six patients were initially pain-free after stimulation. After follow-up, two of the five experienced attacks in periods lasting two to five months and three were almost pain-free (fewer than one attack every three months).

In line with current evidence, we find that after 11 years, our patient showed an 82% decrease in headache attack frequency, from 14 per week to 2.5. Our patient did not respond in the first month of stimulation and underwent 15 programming changes before reaching stable parameters. While the patient continued opioid use along with eletriptan or sumatriptan, these medications in conjunction with DBS were substantially more effective than before electrode implantation. The dose of methadone was doubled four years after DBS implantation, suggesting a potential confounder. To establish a clear connection between DBS and the reported clinical improvements noted by the patient in this case, we rely on three primary indicators. First, the patient reported a reduction in the frequency and intensity of the headaches as well as an improved capability to engage in professional and social activities. Second, the patient had a stable dosing of chronic medications and reduction in the frequency of abortive medications in the years following the DBS implant. Third, the references in the literature show similar improvements in chronic cluster headache symptoms in patients treated with DBS.

Our patient did not experience severe adverse effects after DBS implantation, but Fontaine et al. found serious (subcutaneous infection, preoperative loss of consciousness after test stimulation, and severe micturition syncope) and nonserious (change in libido and hunger and transient diplopia) adverse events in a fraction of their patients [41]. These effects are important considerations for patients requesting this procedure.

We found no studies on the long-term effectiveness of prophylactic pharmacological treatments for chronic cluster headache. However, in the short term, the effectiveness of DBS (50-77% response rate) approaches or exceeds the effectiveness of preventative measures listed by the AHS. A parallel-group study on suboccipital steroid injection reported that after three cortivazol injections, attack frequency was reduced to less than two attacks per day in 95% of treated patients compared to 55% of placebo controls [3]. A double-blind comparison of lithium and verapamil showed over a 50% headache improvement in patients receiving verapamil in the first week compared to only 38% with lithium [4]. A randomized pilot study by Hakim found 48.1% of patients taking warfarin underwent remission compared to 3.1% in placebo after four weeks [5]. After 12 weeks, 66.7% of patients underwent remission on warfarin compared to 22.2% on placebo.

## Conclusions

Mounting evidence demonstrates the effectiveness of DBS as a possible long-term treatment strategy for CCH. We would encourage the consideration of DBS for medically refractory CCH when other treatments have failed. Future randomized controlled trials that incorporate larger sample sizes, extend randomization phases, and offer customized parameters for each patient may yield greater evidence regarding the efficacy of DBS for CCH.
